# Complete Coding Sequence of a Lumpy Skin Disease Virus Strain Isolated during the 2016 Outbreak in Kazakhstan

**DOI:** 10.1128/MRA.01399-19

**Published:** 2020-01-23

**Authors:** Elisabeth Mathijs, Frank Vandenbussche, Meruyert Saduakassova, Tursyn Kabduldanov, Andy Haegeman, Laetitia Aerts, Taskyn Kyzaibayev, Akhmetzhan Sultanov, Steven Van Borm, Kris De Clercq

**Affiliations:** a Sciensano, Unit Exotic Viruses and Particular Diseases, Ukkel, Belgium; b Kazakh Scientific-Research Veterinary Institute LLP, Almaty, Kazakhstan; c Committee for Veterinary Control and Supervision, Ministry of Agriculture of the Republic of Kazakhstan, Nur-Sultan, Kazakhstan; d Republican State Enterprise “National Veterinary Reference Center,” Committee for Veterinary Control and Supervision of the Ministry of Agriculture of the Republic of Kazakhstan, Nur-Sultan, Kazakhstan; Portland State University

## Abstract

Lumpy skin disease virus (LSDV) causes an economically important disease in cattle. Here, we report the complete coding sequence of the LSDV isolate Kubash/KAZ/16, detected in a clinical sample from an infected cow from the outbreak reported on 7 July 2016 in Kazakhstan (Atyrau Region).

## ANNOUNCEMENT

Lumpy skin disease (LSD) is an economically important disease in cattle caused by lumpy skin disease virus (LSDV), a member of the *Capripoxvirus* (CaPV) genus in the *Poxviridae* family. Historically restricted to Africa, the disease reached the Middle East in 2012 and Turkey in 2013. From 2014 to 2015 onward, the disease expanded northwestward to southeast Europe and northeastward, affecting the Caucasus countries the Russian Federation and Kazakhstan (2016) (1). Here, we report the complete coding sequence of an LSDV strain (Kubash/KAZ/16) isolated from the first and only outbreak reported in the Atyrau Region of Kazakhstan. The outbreak was detected on the basis of typical clinical signs of LSD and confirmed by PCR (2).

The DNA was extracted from a skin lesion using the Puregene core kit A (Qiagen) as previously described (3). Presequencing enrichment was performed through 23 PCR amplicons (7,417 to 7,852 bp) with 1-kb overlaps covering the entire genome as described previously (4, 5) using the Q5 high-fidelity DNA polymerase (New England BioLabs). In order to distinguish between the inverted terminal repeats (ITRs), two libraries, each comprising a pool of PCR amplicons corresponding to half of the CaPV genome, were prepared using the Nextera XT DNA library preparation kit (Illumina). Sequencing was performed by the Nucleomics Core (Leuven, Belgium) using a MiSeq benchtop sequencer (reagent kit v3 with 2 × 300-bp paired-end sequencing) to generate 3,303,350 and 1,578,032 paired-end reads per library (mean read length, 216 and 221 nucleotides [nt]). The quality of the data was assessed using FastQC v0.11.3 (http://www.bioinformatics.babraham.ac.uk/projects/fastqc/), and the reads were trimmed using Trim Galore! v0.3.8 (http://www.bioinformatics.babraham.ac.uk/projects/trim_galore/) based on quality (Q score, >30) and length (>80 bp; 5′ clip for R1 and R2, 20 bp). The trimmed reads were assembled *de novo* into a single contig (mean coverage, 133×) using SPAdes v3.9.0 with k values of 21, 33, and 55 and a subset of 20,000 paired-end reads generating the best assembly results (6). The contigs from both libraries were manually merged into a sequence of 150,485 bp, with an average G+C content of 25.89%, evenly distributed. Kubash/KAZ/16 contains a 145,912-bp central coding region flanked by 2 ITRs of at least 2,274 bp containing all expected LSDV open reading frames (ORFs). BLAST results showed that Kubash/KAZ/16 shares 99.99% nucleotide identity with the contemporary LSDV field isolates from Israel (2012; GenBank accession number KX894508) and Greece (2015; KY829023). Annotation and amino acid gene prediction were performed using GATU software relative to the LSDV field isolate Neethling Warmbaths LW sequence (AF409137) (7, 8), and discrepancies were confirmed by Sanger sequencing. A total of 25 nucleotide mutations and 12 single- or 3-nucleotide indels were identified. The localization and the impact of these nucleotide modifications are shown in Table 1. Contemporary LSDV field strain genomes differ by only a couple of mutations. Therefore, additional (nearly) complete genome sequences of circulating LSDV strains are needed to identify a possible source for the outbreak in Kazakhstan.

**TABLE 1:** Nucleotide modifications and their impact on the coding sequence of Kubash/KAZ/16 compared with the LSDV reference strain Neethling Warmbaths LW^*a*^

Gene or IR^*b*^	Nucleotide modification(s)^*c*^	Change in coding sequence
LD005	M	D → N
LD006	M	E → K
IR LD007 to LD008	D	
IR LD009 to LD010	I	
LD010	M	
IR LD010 to LD011	D	
LD013a	D	Frameshift: 328 → 341 aa^*d*^
LD017	M	H → R
IR LD018 to LD019a	I	
LD026a	D, M	Frameshift: 462 → 375 aa
LD028	M	
IR LD029 to LD030	M	
LD035	M	
LD042	M	I → N
LD046	M	
LD059	M	K → F
LD062	M	
LD071	M	E → K
LD075	M	
LD087	M	K → E
IR LD089 to LD090	I	
LD091	M	
LD094	M	S → L
LD096	D	E deletion
LD098	M	
IR LD103 to LD104	I	
IR lD125 to LD126	M	
LD126	M	E → K
LD127	M	
LD128	M	S → F
LD133	M	
IR LD133 to LD134	D	
LD140	M	
IR LD143 to LD144	I	
IR LD146 to LD147	D	
LD148	M	R → K

a GenBank accession number AF409137.

b IR, intergenic region.

c M, mutation; D, deletion; I, insertion.

d aa, amino acid.

### Data availability.

The LSDV isolate Kubash/KAZ/16 sequence has been deposited in GenBank under accession number MN642592, and raw data have been submitted to the SRA under BioProject number PRJNA587601.
